# Supplementary material for: The burden of postpartum depression and its socio-demographic and obstetric correlates among parturient in Bangladesh: A cross-sectional study
Source: PLOS Ment Health. 2025 Sep 23;2(9):e0000443. doi: 10.1371/journal.pmen.0000443 (PMC12798235; doi:10.1371/journal.pmen.0000443)
Supplement: S1 Table — (DOCX) [file pmen.0000443.s001.docx]

**S1Table : Distribution of PPD by Data Collection Site (N=540)**

| Data Collection Site | No PPD n(%) | PPD n(%) | *X^2^* value | P-value |
| --- | --- | --- | --- | --- |
| Medical College Hospital | 83 (45.86%) | 98 (54.14%) |  |  |
| Maternal and Child Hospital | 104 (58.10%) | 75 (41.90%) | 5.44 | 0.06 |
| Upazila Health Complex | 95 (52.78%) | 85 (47.22%) |  |  |
| Total | 282 (52.22%) | 258 (47.78%) |  |  |
