# Supplementary material for: The burden of postpartum depression and its socio-demographic and obstetric correlates among parturient in Bangladesh: A cross-sectional study
Source: PLOS Ment Health. 2025 Sep 23;2(9):e0000443. doi: 10.1371/journal.pmen.0000443 (PMC12798235; doi:10.1371/journal.pmen.0000443)
Supplement: S2 Table — (DOCX) [file pmen.0000443.s002.docx]

**S2 Table: Distribution of participant by EDPS Categories of Depression Severity among different facilities (n=540)**

| Data Collection Site | No Depression n(%) | Possible Depression n(%) | Major Depression n(%) | *X^2^* value | P-value |
| --- | --- | --- | --- | --- | --- |
| Medical College Hospital | 83 (45.86%) | 32 (17.68%) | 66 (36.46%) |  |  |
| Maternal and Child Hospital | 104 (58.10%) | 34 (18.99%) | 41 (22.91%) | 5.44 | 0.06 |
| Upazila Health Complex | 95 (52.78%) | 35 (19.44%) | 50 (27.78%) |  |  |
| Total | 282 (52.22%) | 101 (18.70%) | 157 (29.07%) |  |  |
