# Supplementary material for: The burden of postpartum depression and its socio-demographic and obstetric correlates among parturient in Bangladesh: A cross-sectional study
Source: PLOS Ment Health. 2025 Sep 23;2(9):e0000443. doi: 10.1371/journal.pmen.0000443 (PMC12798235; doi:10.1371/journal.pmen.0000443)
Supplement: Attachment — Submitted filename: Rebuttal Letter.docx [file pmen.0000443.s003.docx]

**Manuscript ID:** PMEN-D-25-00250
**Title:** Strengthening Postpartum Mental Health: Integrating a Mental Health Screening Tool into Routine Postnatal Care

Dear Editors,

Thank you for your previous consideration. I would like to clarify the concern regarding ethical approval and participant recruitment.

While the original manuscript mentioned the study period as January–December 2024, this referred to the full research process, including literature review, protocol development, and report writing.

**Participant recruitment was conducted only after ethical approval**, which was granted on **09 September 2024**. Recruitment occurred from **15 September to 15 October 2024**.

This has now been clearly stated in the revised **Methods section**, and the Human Subjects Research Checklist has been uploaded with this resubmission.

We hope this clarification addresses the concern and respectfully request reconsideration of our manuscript.

Sincerely,
**Dr. Md Foyjul Islam**
On behalf of all co-authors
