# Supplementary material for: The burden of postpartum depression and its socio-demographic and obstetric correlates among parturient in Bangladesh: A cross-sectional study
Source: PLOS Ment Health. 2025 Sep 23;2(9):e0000443. doi: 10.1371/journal.pmen.0000443 (PMC12798235; doi:10.1371/journal.pmen.0000443)
Supplement: Attachment — Submitted filename: Author responses.docx [file pmen.0000443.s004.docx]

PMEN-D-25-00285
Title: Strengthening Postpartum Mental Health: Integrating a Mental Health Screening Tool into Routine Postnatal Care
Journal: PLOS Mental Health

Response to Reviewers

General Statement

We sincerely thank the Academic Editor and the reviewers for their thorough evaluation of our manuscript and for providing valuable feedback that has helped improve the clarity, accuracy, and overall quality of our work. We have carefully addressed all comments and suggestions. Below, we provide our point-by-point responses. Reviewer comments are shown in italics, followed by our responses and details of changes made in the revised manuscript (with line numbers referring to the clean version).

We have submitted:

1. Response to Reviewers (this document)
2. Revised Manuscript with Track Changes
3. Clean Revised Manuscript

Reviewer #1

Comment 1:
What steps, if any, could have been established during your research study period for any woman identified as high risk for postpartum depression (EPDS>13), who does not comply with the recommendation to seek further mental health counselling? Perhaps going the extra step to connect such subjects with an established mental health counsellor would be a valued first-step intervention to help address this issue of strengthening postpartum mental health services in low-income countries like Bangladesh.

Response:
We agree that connecting high-risk women directly with mental health counsellors is an important step for improving follow-up and continuity of care. During the study, women scoring EPDS>13 were counselled on the importance of mental health support, provided with referral information for specialist services, and followed up by phone within one week. Due to logistical constraints and the limited availability of mental health professionals in some settings, direct referral was not always possible. In the revised manuscript, we have added a statement in the Discussion highlighting the potential for integrating direct referral pathways to on-site or tele-mental health counselling as a recommendation for future programmatic integration.
Change made: Discussion, Lines 328-336.

Reviewer #2

Title & Abstract

Comment 1:
Title suggests an implementation study with expected implementation outcomes, but no such outcomes were reported. Consider modifying the title to reflect the actual study.

Response:
We have revised the title to accurately reflect the study design and scope:
Revised Title: The Burden of Postpartum Depression and its Socio-demographic and Obstetric Correlates among Parturient in Bangladesh: A Cross-Sectional Study.
Change made: Title page, Line 3-4.

Comment 2:
Re-write the aim in the abstract to reflect the suggested title.

Response:
The aim in the abstract has been rewritten to:
"To determine the prevalence of postpartum depression (PPD) and its socio-demographic and obstetric correlates among women receiving care at three tiers of health facilities in Bangladesh."
Change made: Abstract, Lines 17–18.

Comment 3:
Insert the 95% CI of the reported prevalence in the abstract.

Response:
The 95% confidence interval for the overall prevalence has been added.
Change made: Abstract, Lines 24-25.

Background

Comment 4:
PPD is a different entity from post-partum psychosis. It is not necessarily correct to say that PPD could turn into post-partum psychosis.

Response:
We have revised the sentence to clarify that while PPD and postpartum psychosis are distinct conditions, severe untreated PPD may increase the risk of later severe mental illness, including psychosis, without implying a direct progression.

Change made: Introduction, Lines-45-50.

Comment 5:
The research gap(s) were not clearly identified.

Response:
We have added a clear statement of the research gap, emphasizing the scarcity of multi-tier, facility-based data on PPD prevalence and correlates in Bangladesh.
Change made: Introduction, Lines 59-73.

Comment 6:
In the penultimate paragraph, making a case for integration of screening of PPD does not arise as the index study has no implementation outcomes.

Response:
We have revised this paragraph to position our findings as baseline evidence that could inform future integration strategies, rather than as a direct evaluation of implementation.
Change made: Introduction, Lines 74-79

Methodology

Comment 7:
Why were women whose babies died or were admitted for severe illnesses excluded?

Response:
These women were excluded because acute bereavement or distress associated with severe neonatal illness could influence EPDS scores independently of postpartum depression, potentially confounding results. We acknowledge that this may limit generalizability and have added this as a limitation in the Discussion.
Change made: Methods, Lines 98-99; Limitations, Lines 363-363.

Comment 8:
Why consider a 5% non-response rate?

Response:
We selected a 5% non-response rate based on prior national maternal health surveys in Bangladesh, which have demonstrated high response rates for face-to-face facility-based interviews.
Change made: Methods, Lines 104-105

Comment 9:
How was the sample size shared across the three tiers of care?

Response:
We recruited an equal number of participants from each of the three tiers of care to ensure balanced representation across different facility levels. This approach allows for direct comparison of postpartum depression prevalence and correlates between primary, secondary, and tertiary healthcare settings without disproportionate weighting toward any one tier.

Change made: Methods, Lines 106-107.

Comment 10:
I doubt if what was described is purposive; it appears more like convenience or consecutive sampling.

Response:
We agree and have revised the term to “consecutive sampling” to accurately describe our recruitment.
Change made: Methods, Line 106.

Comment 11:
Describe the process of translation of the EPDS into the local language.

Response:
We have added details on forward–backward translation, expert panel review, and pilot testing with postpartum women to ensure cultural and linguistic appropriateness.
Change made: Methods, Lines 111-117

Comment 12:
Describe EPDS briefly with emphasis on local psychometric properties.

Response:
We have added a brief description of EPDS, its scoring, and validation evidence from previous Bangladeshi studies.
Change made: Methods, Lines 118-125.

Comment 13:
Insert statistics for inter-rater agreements.

Response:
We have added the Cohen’s kappa coefficient (0.86) obtained from pilot assessments between research assistants.
Change made: Methods, Lines 144-148

Results

Comment 14:
Calculate the 95% CI of the prevalence of PPD reported.

Response:
95% CIs have been added for overall prevalence.
Change made: Results, Lines 201.

Comment 15:
Report prevalence at week 4, 5, 6, …12 among those screened at these points.

Response:
We appreciate the reviewer’s suggestion. However, our study was not designed to stratify postpartum depression prevalence by individual postnatal weeks, and weekly follow-up data were not collected. Therefore, we are unable to report week-specific prevalence at this time.

Discussion

Comment 16:
Comment on differences in prevalence across the three tiers of care.

Response:
We have discussed potential explanations including differences in patient case-mix, referral patterns, and socio-economic characteristics between facility levels.
Change made: Discussion, Lines 325-327

Comment 17:
Exclusion of women with adverse outcomes may violate ethical principle of justice.

Response:
We acknowledge this limitation, noting that these women may be at higher risk and warrant inclusion in future studies with tailored protocols.
Change made: Limitations, Lines 359-371

Comment 18:
Sampling technique may limit generalizability.

Response:
We have added a statement in the limitations section noting that consecutive sampling may limit generalizability to the wider population.
Change made: Limitations, Lines 337-343

Reviewer #3

Comment 1:
Briefly highlight cultural beliefs, stigma, and the role of traditional healers.

Response:
We have added a brief description of cultural beliefs and stigma in Bangladesh, noting that some families may first seek help from traditional healers (kabiraj, ojha) or local village doctors/pharmacy shopkeepers, which can delay access to formal mental health care [Ref: https://doi.org/10.1007/s10597-021-00790-0

Change made: Introduction, Lines 71-73

Comment 2:
Since you've mentioned the expected dropout rate is 5%. But please also mention, exactly how many participants dropped-out of the study/there are missing data.

Response:
Although we anticipated a 5% dropout rate, in practice no participants withdrew during the main study. A total of 27 participants (5% of the calculated sample) were included in the pilot pre-test. Among screen-positive women, follow-up was complete with no missing data. Overall missing data across the study were minimal (<1%) and were addressed using complete case analysis

We hope that the revisions satisfactorily address all reviewer and editor comments. We believe that these changes have significantly improved the clarity, methodological rigour, and contextual relevance of the manuscript. We thank you again for the opportunity to revise and resubmit.
